# A retrospective study of paraganglioma of the urinary bladder and literature review

**DOI:** 10.3389/fsurg.2024.1348737

**Published:** 2024-04-05

**Authors:** Yi Zhao, Zhijun Zhang, ShiJun Wang, Jin Wen, Dong Wang, ZhiGang Ji, YuShi Zhang, HanZhong Li

**Affiliations:** Institution of Urology, Peking Union Medical College Hospital, Peking Union Medical College, Chinese Academy of Medical Sciences, Beijing, China

**Keywords:** paraganglioma of the urinary bladder, diagnosis, transurethral resection of bladder tumor, cystectomy, prognosis

## Abstract

**Objective:**

To review and summarize the characteristics and therapy of paraganglioma of the urinary bladder (PUB).

**Method:**

Patients who underwent the operation in Peking Union Medical College Hospital between January 2012 and December 2021 were reviewed for this retrospective study.

**Results:**

A total of 29 patients, comprising 9 (31%) men and 20 (69%) women, were included. The main manifestations were hypertension, palpitation, and micturition syncope. Eight patients had an increased 24-h urinary catecholamine, and seven of them had increased norepinephrine. Normetanephrine in seven patients was increased. Six of 18 metaiodobenzylguanidine and 8 of 22 octreotide scans were positive. In total, 15 cases underwent laparoscopic partial cystectomy and 14 underwent transurethral resection of bladder tumor. In all patients, the immunohistochemical index of Melan-A, AE1/AE3, and α-inhibin were negative, and chromogranin A, S-100, and succinate dehydrogenase were positive. The Ki-67 of 28/29 cases was under 5%, and 1 case with a Ki-67 of 20% was diagnosed with malignant PUB. A total of 27 patients had a regular follow-up, 2 patients were lost during the follow-up, 3 patients had a recurrence, and 1 of these patients died within 1 year of surgery. The symptoms all disappeared or were relieved after the surgery.

**Conclusion:**

The transurethral surgery approach fits PUB tumors with a size <3 cm or that protrudes into the bladder and can significantly reduce the postoperative hospital stay. Early detection and treatment are effective, and regular review is necessary after the surgery.

## 1 Background

Paraganglioma of the urinary bladder (PUB) is a rare tumor that was first reported by Zimmerman et al. in 1953 (1). Like all the other paragangliomas, PUB derives from chromaffin cells of the sympathetic nervous system. However, it only constitutes 0.06% of urinary tumors and approximately 6%–9.8% of paragangliomas (2, 3). The tumors can be functional or non-functional; the clinical manifestation of the functional tumor mainly includes micturition syncope, hypertension, headache, and palpitation, which are caused by extensively increased endogenous catecholamine (CA) secretions. Due to their rarity and the variable nature of their symptoms, PUBs are commonly misdiagnosed and mistreated. Treatment options generally include transurethral resection or partial or radical cystectomy. We reviewed the clinical and pathological characteristics of all patients diagnosed with PUB in our hospital over the past 10 years and compared postoperative hospitalization and follow-up with different surgical approaches. This will improve our understanding of PUB and help to give the patients a safer and more effective treatment method.

## 2 Methods

We reviewed patients who were diagnosed with PUB and underwent surgery in our hospital between January 2012 and December 2021. The patients’ general information, laboratory and radiology examinations, surgery, pathology, and follow-ups were collected. All PUB tumors were defined as functional when urine or plasma fractionated CAs or fractionated or total metanephrines (MN) were elevated above the upper limit of respective reference ranges. Length of stay exceeding the 75th percentile of the total length of stay was defined as an extended length of stay (4). High-performance liquid chromatography with electrochemical detection determined 24-h urinary CA levels in all patients. In addition, normetanephrine (NMN) and MN were detected in blood samples utilizing the same method for patients from 2019 onward, which helped us to save diagnostic time and improve accuracy. The operative method of PUB was diverse. According to our experience, when the tumor diameter is ≤3 cm, a transurethral resection of bladder tumor (TURBT) can be selected, which can ensure the *en bloc* resection of the tumor and complete removal from the urethra to avoid implantation and metastasis. All tumors were completely resected. All surgical specimens were diagnosed by urologic pathologists. Tumor markers for paragangliomas of the bladder, including CD56, NSE, chromogranin A (CgA), Syn vimentin, succinate dehydrogenase (SDHB), Von Hippel–Lindau (VHL), PGP9.5, and S-100 protein, were detected using immunohistochemical techniques. Tumor size was determined based on the largest diameter of the PUB on histopathology. To assess the effectiveness of PUB treatment, a long-term follow-up was carried out by reviewing patients in the outpatient clinic or by telephone interviews at intervals of approximately 3–6 months. The follow-up period is calculated from the date of surgery to the date of the last follow-up or death. SPSS version 26.0 software was used for data processing and analysis. In this study, measurement data that did not obey normal distribution were expressed as median (M) and quartiles (P25–P75), and the Mann–Whitney *U* test was used for comparison between groups. Count data were expressed by frequency or composition ratio, and the chi-square test was used for comparison between groups. *P* < 0.05 was considered statistically significant.

## 3 Results

### 3.1 Patient demographics

In this study, 29 cases of PUB were diagnosed, accounting for 5.7% (29/508) of patients with all paragangliomas treated in our hospital during the same period. Baseline characteristics, diagnostic findings, operation methods, results, and postoperative follow-up for each patient can be found in Table 1. The mean age of the patients with PUB was 48 years (range 28–68), and these included 9 (31%) men and 20 (69%) women. Of the 29 cases, 11 (37.9%) were functional and 18 (62.1%) were non-functional. Patients with PUB presented with strong headache (44.9%), palpitation (62.1%), weakness (20.7%), and increasing blood pressure after urination (41.4%). Two patients (6.9%) detected the tumor coincidentally, without any symptoms. One patient with a history of hypertension for more than 10 years had postoperative symptom relief and his blood pressure remained at normal levels during the long-term follow-up. Among the 20 patients who underwent SDHB gene screening for paraganglioma genetic syndrome, four patients were identified as SDHB-positive (SDHB+), one patient was classified as SDHB-indeterminate (SDHB±), and the remaining patients tested negative for SDHB (SDHB–). All patients experienced complete symptom relief after surgery, with a recurrence rate of 20% observed in SDHB+ or SDHB± patients, while no recurrences were observed in those who tested negative for SDHB.

**Table 1:** Baseline characteristics, operation methods, results, diagnosis, and follow-up for each patient.

Number	Gender	Age	Tumor size	Operation methods	Results	Diagnosis	Follow-up
1	M	39	2.7	Partial cystectomy	Asymptomatic	PUB	Recurrence
2	F	49	0.9	TURBT	Non-remission	PUB	Relief after 1 year
3	F	50	4.7	Partial cystectomy	Remission	PUB	Normal
4	F	51	1.8	TURBT	Remission	PUB	Normal
5	M	34	2.7	TURBT	Remission	PUB	Normal
6	F	68	2.4	Partial cystectomy	Remission	PUB	Normal
7	F	44	1.1	Partial cystectomy	Remission	PUB	Normal
8	F	43	8.8	Partial cystectomy	Remission	PUB	Recurrence
9	M	51	3.2	Partial cystectomy	Remission	PUB	Normal
10	F	54	2.0	TURBT	Remission	PUB	Normal
11	M	50	1.5	TURBT	Remission	PUB	Normal
12	F	36	2.0	Partial cystectomy	Remission	PUB	Normal
13	F	61	1.5	TURBT	Remission	PUB	Normal
14	F	40	3.5	Partial cystectomy	Remission	PUB	/
15	F	46	1.4	TURBT	Remission	PUB	Normal
16	F	56	2.0	Partial cystectomy	Remission	PUB	Normal
17	M	49	6.5	Partial cystectomy	Remission	PUB	Recurrence
18	F	40	1.2	Partial cystectomy	Remission	PUB	Normal
19	F	50	1.7	TURBT	Remission	PUB	Normal
20	M	62	1.1	TURBT	Remission	PUB	Normal
21	M	28	2.9	Partial cystectomy	Asymptomatic	PUB	Normal
22	F	47	1.1	TURBT	Remission	PUB	Normal
23	F	28	3.0	Partial cystectomy	Remission	PUB	Normal
24	M	61	2.7	TURBT	Remission	PUB	Normal
25	F	48	2.0	TURBT	Remission	PUB	Normal
26	F	55	4.0	Partial cystectomy	Remission	PUB	Normal
27	M	56	2.7	Partial cystectomy	Remission	PUB	Normal
28	F	39	1.6	TURBT	Remission	PUB	/
29	F	47	1.8	TURBT	Remission	PUB	Normal

“/” indicates missing follow-up data.

### 3.2 Treatment

**Figure 1:**
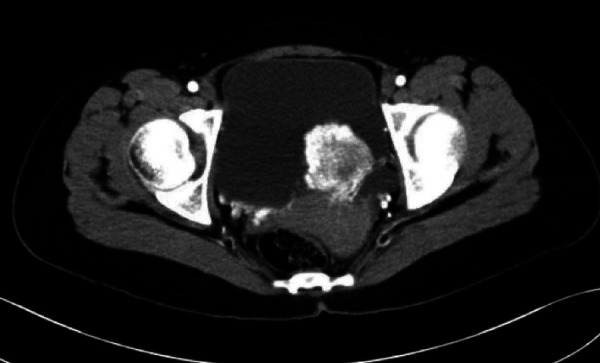
The arterial phase enhanced CT scan of the patient whose PUB tumor was located in left posterior wall.

**Figure 2:**
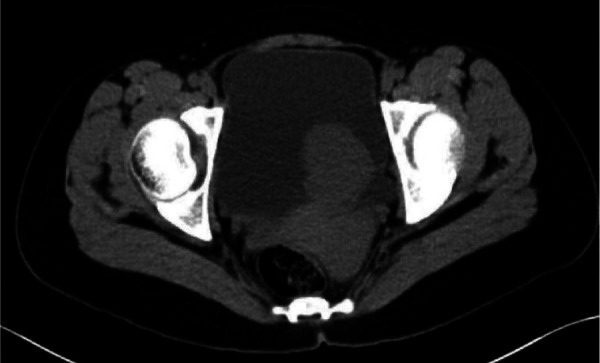
The unenhanced CT scan of the patient whose PUB tumor was located in left posterior wall.

**Figure 3:**
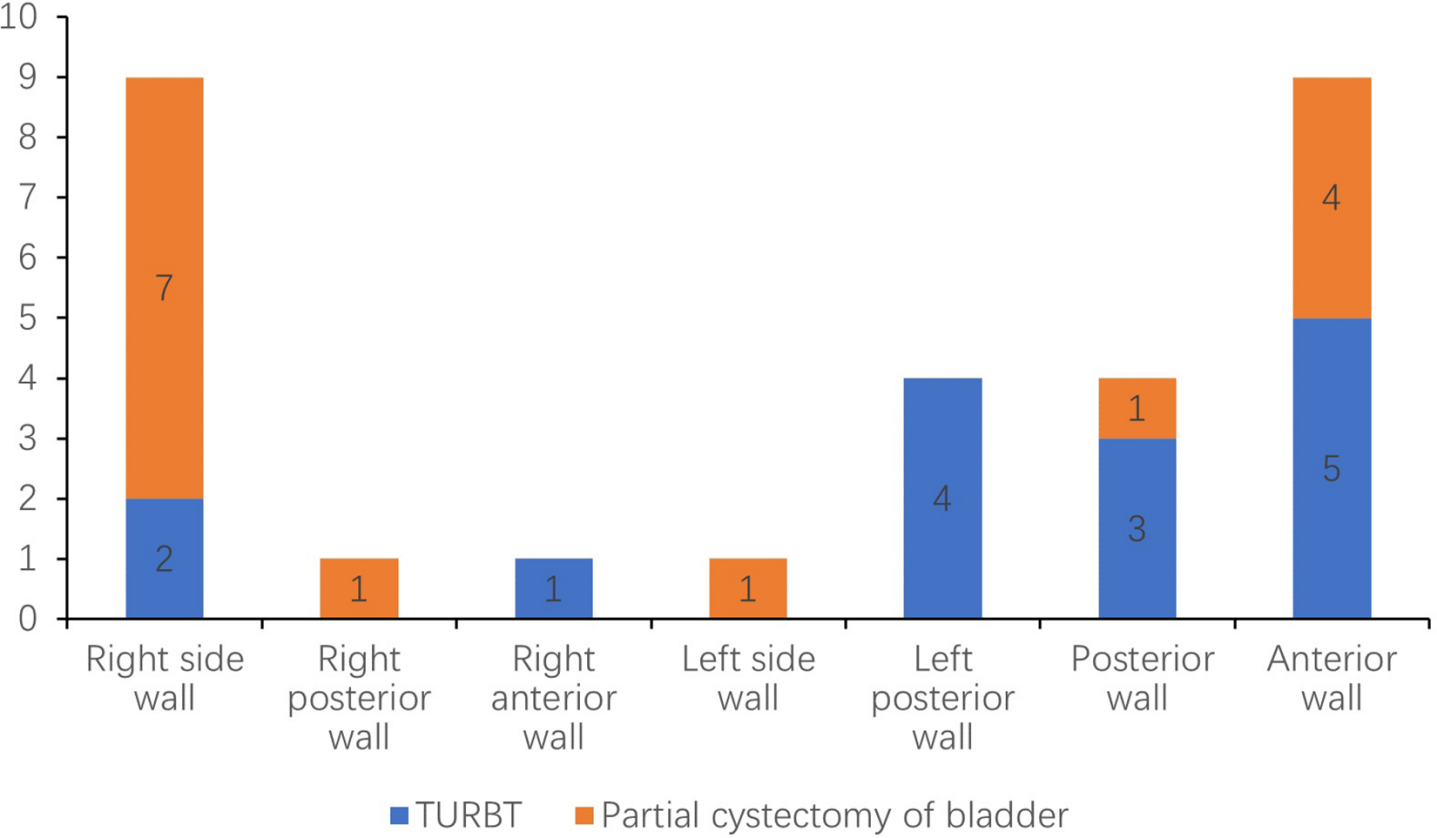
The distribution of different tumor locations of bladder and surgical approach.

The traditional methods of B-ultrasound and contrast-enhanced enhanced computed tomography (CT) were taken preoperatively to assess the location and the statement of invasion of the tumor (Figures 1, 2). PUB performs an obvious enhancement because of the rich blood supplement. The mean size of the largest tumor diameter was 2.57 cm. In total, 18 patients underwent metaiodobenzylguanidine (MIBG) imaging and 6 (33.3%) patients had positive results. A total of 22 patients underwent octreotide imaging, and 8 (36.3%) patients had positive results. MIBG and octreotide imaging were both positive in two patients. Notably, two patients had positive MIBG imaging results but negative octreotide imaging results, and one patient had negative MIBG imaging results but positive octreotide imaging results. Three patients underwent 68GA PET-CT for suspected metastases and all had positive results. The locations of the PUBs included the right wall (9 cases, 31.0%), right posterior wall (1 case, 3.4%), right anterior wall (1 case, 3.4%), left wall (1 case, 3.4%), left posterior wall (4 cases, 13.8%), posterior wall (4 cases, 13.8%), and anterior wall (9 cases, 31.0%) (Figure 3).

**Figure 4:**
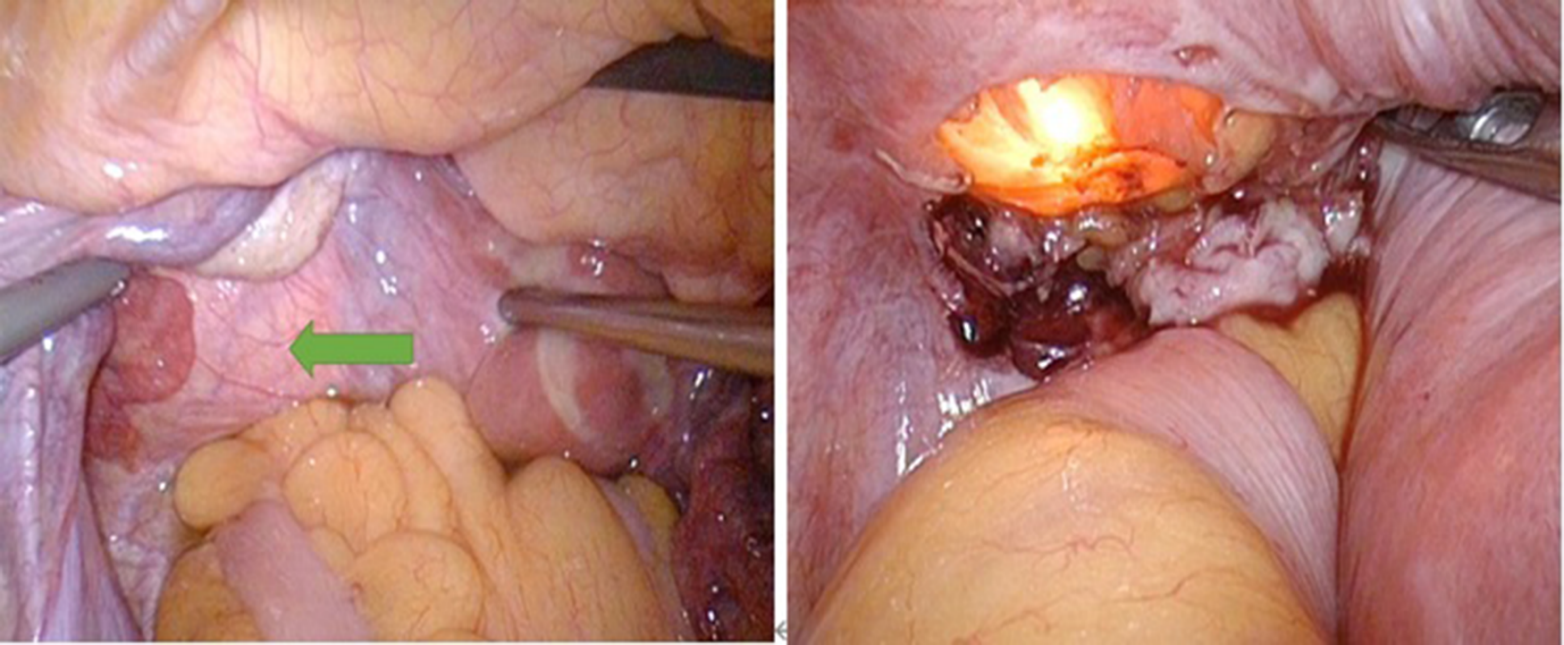
In a combined transurethral laparoscopic partial cystectomy, the transurethral approach helps the surgeon avoid damaging important anatomical structures.

All 29 patients underwent rigorous preoperative preparation using alpha blockers for 2–4 weeks. The preoperative evaluation of patients’ supine blood pressure, heart rate, hematocrit (HCT), and other indicators suggested that surgery should be performed only after full preoperative preparation and that changes in blood pressure and heart rate should be strictly monitored during the operation. The results showed that the blood pressure and heart rate of all patients did not fluctuate significantly, and the hemodynamics were stable. All patients underwent surgery in our hospital, with 15 combined transurethral and laparoscopic partial cystectomies (Figure 4) and 14 TURBT. Among the patients who underwent a combined transurethral and laparoscopic partial cystectomy, the tumor size in eight cases was smaller than 3 cm; all patients who underwent a TURBT procedure had a tumor size smaller than 3 cm. The mean postoperative hospital stay was 7.7 days in the partial cystectomy group and 3.6 days in the TURBT group, with a significant difference in whether postoperative hospital stay was prolonged in the two groups (*p* < 0.001) and no difference in whether there was a recurrence after surgery (*p* > 0.05) (Table 2).

**Table 2:** Intergroup comparison of postoperative hospital stay by surgical approach.

Index		Partial cystectomy group	TURBT group	*Z*/*χ*^2^	*P*
Age		46 (39.55)	50 (47.56)	−1.027	0.304
Gender	Male	5 (33.3%)	4 (28.6%)	−0.272	0.785
	Female	10 (66.7%)	10 (71.4%)		
Postoperative hospital stay		7.7 (6.9)	3.6 (3.5)	−4.232	<0.001
Prolonged postoperative hospital stay	Extension	12 (80%)	0 (0%)	19.106	<0.001
	No extension	3 (20%)	14 (100%)		
Postoperative recurrence	Recurrence	3 (21.4%)	0 (0%)	1.340	0.247
	No recurrence	11 (78.6%)	13(100%)		

### 3.3 Pathology

**Figure 5:**
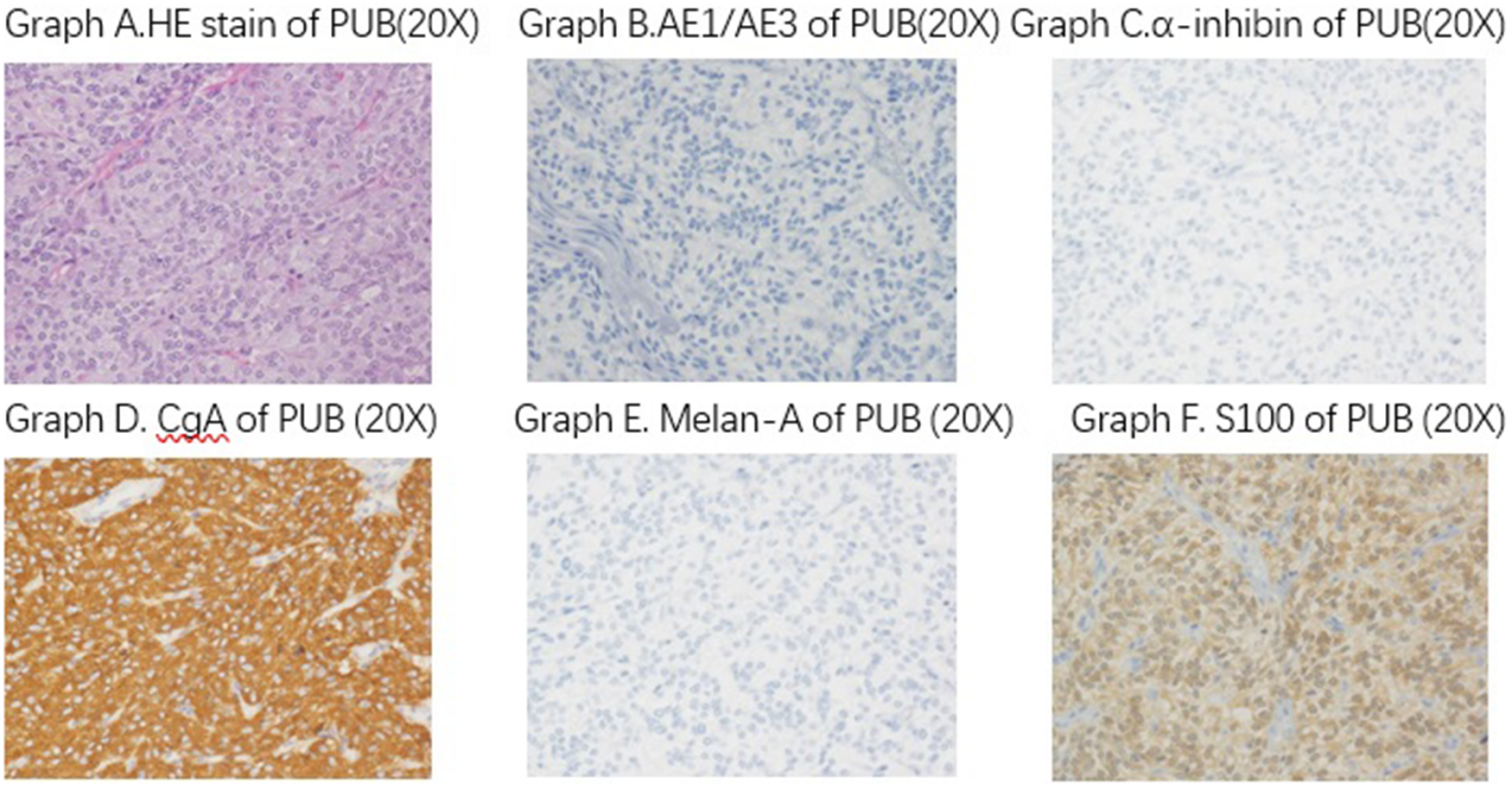
The pathology historical results of the 29 patients are all paraganglioma of urinary bladder. The immunohistochemical index of Melan-A, AE1/AE3, and α-inhibin are negative, while CgA, S-100, and SDHB are positive.

The historical pathology results of the 29 patients were all paraganglioma of urinary bladder. The immunohistochemical index of Melan-A, AE1/AE3, and α-inhibin were negative, while CgA, S-100, and SDHB were positive. The Ki-67 index can determine the proliferative activity of the tumor. In our patients, the Ki-67 index of 28 cases was <5%. One patient, whose Ki-67 was 20%, was diagnosed with metastatic PUB, and the pathology results showed that the bladder muscle was invaded and an intravascular tumor thrombus had formed (Figure 5).

### 3.4 Follow-up and prognosis

Of the 29 cases, 2 (6.9%) were lost during the follow-up. The remaining 27 patients were reviewed regularly. One patient had a new mass in the left pelvis 9 months after surgery; metastases in the lumbosacral region, the right side of the chest, and the right side of the back were detected by MIBG and octreotide scanning. The patient died within 1 year after surgery. The patient with a recurrence 2 years after surgery showed intravesical metastases, right paracolonic parappendiceal metastases, multiple metastases in both lungs (the largest in the lower lobe of the left lung), and an elevated systolic blood pressure of >180 mmHg. We gave him alpha receptor blockers to control the blood pressure, and there was no progress with the recurred mass and multiple metastatic foci. One patient had a recurrence 5 years postoperatively, with two masses detected: one in the retroperitoneum and the other in front of the sacrum. Temozolomide was taken with side effects of weakness and nausea. The symptoms of headache, palpitation, and high blood pressure after urination in 26 patients disappeared after the surgery, and one patient experienced symptom relief within 1 year.

## 4 Discussion

A paraganglioma is a non-epithelial tumor that arises in a paraganglial location. Less than 10% of paragangliomas are found in the urinary bladder, and young people are more likely to develop PUB. PUBs arise more frequently in the trigone of the bladder, with a mean size of 3.9 cm (5). To the best of our knowledge, we have reported the largest number of patients with PUB in one center to date.

The detection and diagnosis of PUB in the early stage depend on the clinical manifestation of hypertension and radiology examination. Symptoms may include headache, paranesthesia, dyspnea, angina, hematuria, and lower urinary tract symptoms (6). Unfortunately, PUB is always misdiagnosed as bladder cancer, especially the non-functional tumors with no symptoms. According to our previous literature review, 61.6% of patients with PUB were misdiagnosed before pathologic diagnosis, and less than 30% were diagnosed preoperatively (7).

Due to the difficulties in diagnosing PUB, crucial laboratory examinations and imaging analyses are necessary before surgery. CAs, which are secreted by chromaffin cells, include dopamine, adrenaline, and norepinephrine, and are important indexes. The level of these indexes, either in blood or 24-h urinary samples, are increased in functional PUB, and these laboratory results can help us finish the etiology diagnosis before the operation. In our study, all three patients with non-functional PUB had a normal level of 24-h urine CA, NMN, and MN. Almost all abnormal laboratory indexes show increased NE and NMN in functional PUB tumors. This may suggest that increased NE and NMN are specific to PUB tumors.

Imaging analysis provides a localization diagnosis and improves the etiology diagnosis. Contrast-enhanced CT and magnetic resonance imaging (MRI) are two basic screening methods. PUB demonstrates a regular-shaped bladder tumor with obvious enhancement and hyperintensity on T2-weighted imaging (T2WI) (8). MIBG and octreotide imaging are used as important tools in the diagnosis of PUB; in some studies, MIBG imaging has shown a higher sensitivity and specificity and is superior to octreotide imaging, but the latter has a clear advantage in the detection of some metastatic lesions. In our study, three patients had recurrent postoperative metastases: one patient underwent MIBG imaging with positive results; one patient underwent octreotide imaging with positive results; and one patient had both scans, in which MIBG imaging was negative and octreotide imaging was positive. This is consistent with results in previous studies. For the patients who underwent both MIBG and octreotide imaging, one was negative on MIBG imaging but positive on octreotide imaging, and two were positive on MIBG imaging but negative on octreotide imaging. Overall, MIBG and octreotide scans each have their own advantages and are complementary, which need to be selected with the patient's characteristics in the clinic. In previous studies, fluorodeoxyglucose (FDG)-PET was found to be more sensitive than MIBG imaging (9), whereas Ga-68 DOTATATE PET/CT could detect metastatic PUBs (10). However, non-functional PUBs are difficult to detect preoperatively because of the lack of secreting CA and non-typical symptoms. A case of functional PUB was reported without any radiographic and laboratory tests (11). Musa et al. (12) recommended cystoscopy before surgery because PUBs have the cystoscopic feature of hypervascularization. However, the final diagnosis must be based on histopathology and immunohistopathology after tumor resection.

Surgery is the most important treatment for PUB. To date, two main surgical options, transurethral resection and combined transurethral and laparoscopic partial cystectomy, have been used. Most PUB tumors were functional and could be detected in the early stage; the tumor size was small, and transurethral resection was a safer and better surgical approach. Approximately one-fifth of patients were treated with the TURBT procedure alone (13). The combined transurethral and laparoscopic partial cystectomy should be used for tumors that invade the muscle layer of the bladder or go even deeper. Transurethral methods can help surgeons avoid injury of this important anatomical structure, such as the bilateral ureteral orifice. We can coagulate the vessel at the tumor base early and it may be beneficial to use short bursts to limit the fluctuations in blood pressure during the procedure (14). At present, laser resection and electro-excision are reported to treat PUB, with good results (15), while it is suggested that resection rarely leads to a high level of recurrence (16).

In this article, we compared the effects of both partial cystectomy and TURBT procedure on postoperative length of stay and postoperative recurrence. The results showed that the different surgical approaches did not have an effect on postoperative recurrence and that the TURBT procedure significantly reduced the postoperative length of hospital stay. Moreover, recent findings suggest that the TURBT procedure is feasible for tumors with a diameter <3 cm with adequate preoperative preparation (17). Therefore, we recommend the TURBT procedure for PUBs smaller than 3 cm, while for larger tumors, partial cystectomy or radical cystectomy can be chosen, depending on the PUB’s invasion of the bladder wall. Pelvic lymph node dissection or biopsy is necessary if metastasis is suspected. However, due to limitations in our sample size, we were not able to compare the differences between the two surgical approaches separately when the tumor was smaller than 3 cm. Therefore, more studies are needed to confirm this conclusion. On the other hand, almost all paragangliomas have a whole regular membrane, and excision extension involving the muscularis of the bladder is the key point to respect the tumor completely, and complete excision of the membrane is most important to avoid or decrease the rate of recurrence.

Pathology is the gold standard for a definite diagnosis. A typical paraganglioma has neuroendocrine markers combined with neuroendocrine markers and negative mesenchymal and epithelial markers. CD56, NSE, CgA, Syn vimentin, SDHB, VHL, PGP9.5, and S-100 protein are in common use (18). In our study, a typical PUB tested negative for Melan-A, α-inhibin, and AE1/AE3, and positive for CgA, S-100, and SDHB. A Ki-67 index >5% indicates a high risk of metastasis. Genetic disorder is another factor in occurrence, and SDHB is the most common gene associated with the highest rate of metastasis (19).

## 5 Conclusion

PUB is a rare bladder tumor with gradually advanced appropriate methods of diagnosis and surgery approaches in recent years. The transurethral surgery approach fits for most PUB tumors with a size <3 cm or that protrudes into the bladder. Early detection and treatment are effective, and regular postoperative reviews are necessary.

## Data Availability

The raw data supporting the conclusions of this article will be made available by the authors, without undue reservation.
