# Corrigendum: A retrospective study of paraganglioma of the urinary bladder and literature review

**DOI:** 10.3389/fsurg.2025.1556161

**Published:** 2025-03-04

**Authors:** Yi Zhao, Zhijun Zhang, ShiJun Wang, Jin Wen, Dong Wang, ZhiGang Ji, YuShi Zhang, HanZhong Li

**Affiliations:** Institution of Urology, Peking Union Medical College Hospital, Peking Union Medical College, Chinese Academy of Medical Sciences, Beijing, China

**Keywords:** paraganglioma of the urinary bladder, diagnosis, transurethral resection of bladder tumor, cystectomy, prognosis

**Corrigendum on**
A retrospective study of paraganglioma of the urinary bladder and literature review By Zhao Y, Zhang Z, Wang S, Wen J, Wang D, Ji Z, Zhang Y and Li H (2024). Front Surg. 11:1348737. doi: 10.3389/fsurg.2024.1348737

**Incorrect Affiliation**

In the published article, there was an error in affiliation [1]. Instead of “[Institution of Urology, Peking Union Medical College Hospital, Chinese Academy of Medical Sciences, Beijing, China]”, it should be “[Institution of Urology, Peking Union Medical College Hospital, Peking Union Medical College, Chinese Academy of Medical Sciences, Beijing, China]”.

The authors apologize for this error and state that this does not change the scientific conclusions of the article in any way. The original article has been updated.

